# Change of sagittal spinal alignment and its association with pain and function after lumbar surgery augmented with an interspinous implant

**DOI:** 10.1186/s13013-017-0109-z

**Published:** 2017-01-30

**Authors:** Rebecca J. Crawford, Quentin J. Malone, Roger I. Price

**Affiliations:** 1 Institute for Health Sciences, School of Health Professions, Zürich University of Applied Sciences, Technikumstrasse 81, Winterthur, CH-8401 Switzerland; 2 Faculty of Health and Exercise Sciences, Curtin University, Perth, Australia; 3 Centre for Neurological Surgery, Perth, Australia; 4 Department of Medical Technology and Physics, Sir Charles Gairdner Hospital, Perth, Australia; 5 School of Physics, University of Western Australia, Perth, Australia

**Keywords:** Interspinous implant, DIAM, Spinal alignment, Low back pain, Clinical outcomes, Radiological outcomes

## Abstract

**Background:**

Interspinous spacer/implants like the Device for Intervertebral Assisted Motion (DIAM™) are controversially yet commonly used in the surgical treatment of lumbar degenerative pathologies. Criticism is based on ill-defined indications, lack of superiority over decompression, and a poorly understood mechanical effect. Yet, continued use by surgeons implies their perceived clinical merit. We examined radiographic spinal alignment for 12 months, and pain and function for 24 months, after DIAM-augmented surgery to improve the understanding of the mechanical effect relating to clinical outcomes in patients.

**Methods:**

We undertook a single-surgeon prospective, longitudinal study of 40 patients (20 F, 20 M) who received DIAM-augmented surgery in treatment of their symptomatic lumbar degenerative condition. Outcomes measured included sagittal spinal alignment (lumbar lordosis, sacral inclination, primary (PDA), supradjacent (SDA) disc angles, and regional sagittal balance (RSB; standing lateral radiographs), and back and leg pain (visual analogue scale; VAS) and function (Oswestry Disability Index; ODI). Responders were identified as those with clinically meaningful improvement to pain (>20%) and function (>15%) at 24 months postoperatively; features of sagittal spinal alignment between responders and non-responders were examined.

**Results:**

Sagittal alignment was unchanged at 12 months. At 6 weeks postoperatively, PDA (mean (SD)) reduced by 2.2° (4.0°; *p* < 0.01) and more-so in back pain non-responders (3.8° (3.2°)) than responders (0.7° (4.4°); *p* < 0.05). Positive preoperative RSB in responders (26.7Rmm (42.3Rmm); Rmm is a system-relative measure) decreased at 6 weeks (by 3.1Rmm (9.1Rmm)). Non-responders had a negative RSB preoperatively (−1.0Rmm (32.0Rmm)) and increased at 6 weeks (11.2Rmm (15.5Rmm); *p* < 0.05). Clinically meaningful improvement for the whole cohort for back pain and function were observed to 24 months (back pain: 25.0% (28.0); function: 15.4% (17.6); both *p* < 0.0001).

**Conclusions:**

Unaltered sagittal alignment at 12 months was not related to symptoms after DIAM-augmented lumbar surgery. Subtle early flattening at the index disc angle was not maintained. Preoperative and early post-operative sagittal alignment may indicate response after DIAM-augmented surgery for mixed lumbar pathologies. Further investigation toward defining indications and patient suitability is warranted.

## Background

Interspinous implants are controversially used in the surgical treatment of lumbar degenerative pathologies [1]. An inadequate etiological understanding, ill-defined indications, and lack of superiority compared to more cost-effective decompression, have drawn their utility into question [2–6]. Second generation and perhaps the most commonly used and investigated interspinous implants include the X-Stop™ [7], Wallis™ [8], DIAM™ [9], and Coflex™ [10] devices, which provide a non-fusion surgical option in the treatment of lumbar segment disease [11]. These spacer devices vary in design and employ compressible (DIAM and Wallis) or rigid (X-Stop and Coflex) composite materials; they are surgically introduced into the interspinous space using differential access and insertion techniques that aim toward closest approximation to the deep spinous process and laminae in order to induce distraction of the posterior elements [11]. An increasing number of interspinous devices are available and appear to have wide international adoption, with promising new evidence emerging for their benefit in discreet diagnoses and indications [12, 13]. Interspinous implants may have relevance in safely ameliorating back and associated leg pain in an ageing society where co-morbidities necessitate minimised invasive interventions. Identifying features of patients with superior clinical outcomes is fundamental to optimising the successful application of these devices.

For the purposes of this paper examining a single device, and in light of the vast literature dedicated to the many individual interspinous implants, we subsequently refer to studies reporting the Device for Intervertebral Motion (DIAM; Medtronic Sofamor Danek, Memphis, USA). The DIAM is an X-shaped elastomeric interspinous spacer with developer-purported indications including lumbar spinal stenosis, herniated and degenerated disc, facet joint pain syndrome and minor degenerative spondylolisthesis [9]. Low lumbar implantation predominates in augmenting decompression [5, 6, 14–17]. Evidence reports meaningful reduction in pain and function [9, 15–17], yet effect can be variable. Few studies examine the relationship between pain and function, and sagittal spinal alignment as related determinants of post-operative outcome after surgeries involving DIAM implantation [16, 18, 19].

Developer guidelines for the application of the DIAM [9] direct that the largest possible device be implanted to optimise therapeutic effect by maximally-tensioning the supraspinous ligament without imposing actual segmental kyphosis. Subtle relative kyphosis at the index segment has been shown with DIAM in cadaveric spines [20, 21], and in the early postoperative period in patients [16, 18]. However, no studies that we are aware of have attempted to identify pre- or post-operative skeletal features of sagittal alignment that may be prognostic for successful patient-reported outcomes. We therefore evaluated the effect in-vivo on sagittal spinal alignment of DIAM-augmented surgery at regional, operated, and supradjacent levels, and in relation to clinically meaningful improvement in self-reported pain and function.

## Methods

### Study overview

This study assessed 40 patients (mean age: 54 years; SD: 13 years) including 20 women (mean age: 56 years; SD: 9 years), and 20 men (mean age: 51 years; SD: 14 years) from a larger prospective, longitudinal, effectiveness investigation examining clinical outcomes for 2 years after DIAM-augmented lumbar surgery from a single-surgeon practice [17]. Outcome measures included patient-reported pain and function, in addition to measures of skeletal spinal curvature using standing lateral radiography, respectively. The study received institutional ethics and review board approvals from the University of Western Australia and complied with the Declaration of Helsinki. Patients were consecutively recruited with informed consent after the surgical author (QM) made the clinical decision for DIAM-augmented surgery. Patients with previous lumbar spinal surgery and an inability to communicate in English (for completion of questionnaires) were excluded. The 40 patients examined for the radiographic analysis described in the present paper were all of the subjects from the larger study that had complete and usable radiographs. Preoperative questionnaires and radiographs were completed within 1 week of surgery; postoperative radiographs were undertaken at 6 weeks and 12 months, and questionnaires at 6 weeks, 12 months and 24 months.

The surgeon’s (QM) preoperative diagnoses of lumbar spinal stenosis (*n* = 27), facet joint pain syndrome (*n* = 3), and minor degenerative spondylolisthesis (*n* = 10) were based on patient case-notes, clinical examinations, static and functional imaging, facet joint injections, diagnostic blocks, and/or discography (as relevant). Intended vertebral levels, number of implants, and the primary (in case of multiple) index segment were recorded preoperatively by the surgical author (QM), and verified postoperatively via case-note audit (by author RJC). All DIAMs were implanted at or caudal to L3/4. Twenty-two patients received a single DIAM, 15 had two implants, and three received three devices. The primary (index) level for the implant was L4/5 (*n* = 26), L5/S1 (*n* = 12) and L3/4 (*n* = 2).

### Surgical procedure

Subjects underwent DIAM-augmented lumbar surgery according to routine supraspinous ligament-sparing procedure for the device without ligatures [9]. Briefly regarding device implantation, a mid-sagittal incision is made and muscle tissue retracted from the spinous process bilaterally, the interspinous space is prepared approximating the laminae as close as possible, with subsequent excision of interspinous ligamentous tissue and applied distraction to optimise the size of implanted device, and then employing device-specific instruments, the DIAM is inserted from one side.

### Pain and function

Evaluations of patient-reported back and leg pain, and function, were made using visual analogue scales (VAS) [22] and Oswestry Disability Index (ODI) [23], respectively.

### Responder analysis

Within-subject absolute changes for back pain, leg pain, and function between 12 months and preoperative time-points were used to establish outcome at 1 year after surgery. Subjects were categorised into three groups [moderate-, minimal-, non-responders] based on definitions for minimal clinically important differences [24]. Improvement was deemed moderate with >30% reduction in ODI or VAS, minimal 15–29% ODI and 20–29% VAS, and non-response ODI <15% and pain <20%.

### Radiographic evaluation

**Fig. 1:**
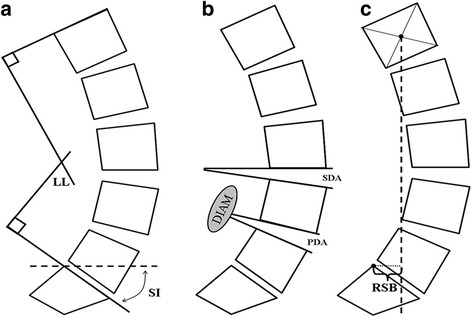
Schematic representation of the methods employed to measure lumbar sagittal alignment from standing lateral radiographs showing: (**a**) Lumbar lordosis and sacral inclination; (**b**) Primary and supradjacent disc angle; and (**c**) Regional sagittal balance. Footnote: *LL* lumbar lordosis, *SI* sacral inclination, *PDA* primary disc angle, *SDA* supradjacent disc angle, and *RSB* regional sagittal balance

The standardised procedure for lateral views involved: 100 cm film-tube distance, centred at L3, and barefoot standing in the clavicle position (elbows flexed with lightly clenched fists resting over their ipsilateral clavicles) to optimise visualisation of lumbar vertebral landmarks [25]. Lumbar skeletal alignment was measured from digital [JPEG, 1200dpi] images using a bespoke programme. Vertebral bodies (L1-S1) were defined with a standardised four-point quadrilateral system based on an established method [26]. Computed radiographic variables included: lumbar lordosis (referencing superior end-plates of L1 and S1) [27]; sacral inclination (referencing superior end-plate of S1 and the horizontal); disc angles (referencing the inferior end-plate of the upper vertebra and superior end-plate of the lower vertebra) PDA and SDA; and RSB defined as the horizontal distance between plumb-line from the centroid of L1,and the posterior corner of the S1 superior end-plate [28]. Radiographic methods are schematically illustrated in Fig. 1.

Correction for magnification between serial radiographs was necessary for the non-angular variable RSB. Projected areas of the L2, L3 and L4 vertebral bodies enabled correction-factor calculation. Ratios between postoperative and preoperative images were scaled according to the square root of the combined L2-4 vertebral body areas of the baseline image. The magnification metric was unknown so RSB is denoted by system-relative millimetres (Rmm).

### Statistical analysis

Data were analysed using Microsoft Excel and StatView (Abacus Concepts, Berkley, 1992). Descriptive statistics trichotimised by responder category (moderate, minimal or non) reported mean (±SD) lumbar lordosis, sacral inclination, PDA, SDA, and RSB at baseline, and change scores between baseline and 6 weeks. Change was assessed according to response and categorisation using unpaired t-tests. Repeated measures ANOVA with Scheffe’s post-hoc tested serial change. Box-plots present data indicating 10th, 25th, 50th, 75th and 90th percentiles. Association between radiographic variables, pain and function were tested using Pearson’s correlation coefficient [r]. Statistical significance was *p* < 0.05.

### Repeatability of the measurement technique

Intrarater repeatability of radiographic data was assessed with repeated measurements (1 week apart) of ten baseline images (Table 1). No significant difference for any variable was noted but was lowest for PDA compared to the other variables.

**Table 1:** Repeat measurements (mean (SD)) of the same baseline lateral radiographs for ten surgical cases

	Test 1	Test 2	Difference	2-tail *t*-test
Lumbar Lordosis (°)	57.1 (11.4)	57.0 (12.2)	−0.15 (2.32)	*p* = 0.86
Sacral Inclination (°)	37.4 (5.2)	37.3 (6.2)	0.07 (1.56)	*p* = 0.90
Primary Disc Angle (°)	15.6 (6.8)	16.1 (7.5)	0.50 (1.09)	*p* = 0.20
Regional Sagittal Balance (Rmm)	2.3 (27.9)	2.0 (27.5)	0.31 (1.92)	*p* = 0.62

## Results

**Fig. 2:**
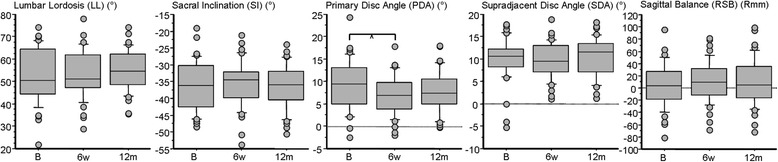
Box-plots with outliers revealing serial change in lumbar lordosis, sacral inclination, primary disc angle, supradjacent disc angle and regional sagittal balance. Footnote: *B* preoperative baseline, *6w* 6 week time-point, *12 m* 12 month time-point, *LL* lumbar lordosis, *SI* sacral inclination, *PDA* primary disc angle, *SDA* supradjacent disc angle, and *RSB* regional sagittal balance; * = *p* < 0.01

**Fig. 3:**
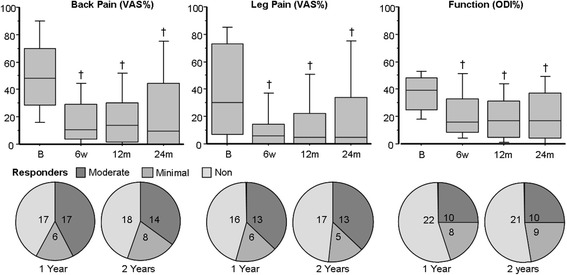
Box-plots (*top*) and pie-charts (*bottom*) revealing patient-reported change in back pain, leg pain and function for 40 cases out to 2 years after DIAM-augmented surgery. Box-plots reveal serial change with significant differences compared to baseline values noted at each time-point. Pie-charts indicate the number of cases in responder groups for each measure at 1 and 2 years postoperatively. Footnote: *B* preoperative baseline, *6w* 6 week time-point, *12 m* 12 month time-point, *LL* lumbar lordosis, *SI* sacral inclination, *PDA* primary disc angle, *SDA* supradjacent disc angle, and *RSB* regional sagittal balance; * = *p* < 0.0001

No change in lumbar lordosis, sacral inclination, SDA, or RSB was shown at either 6 weeks or 12 months. PDA flattened by 2.2° (4.0°; *p* < 0.01) at 6 weeks, and remained 1.5° (4.7°) flatter at 12 months (not significant) (see Table 2 and Fig. 2). Back pain, leg pain and function meaningfully improved at all post-operative time-points (*p* < 0.0001; Table 2 and Fig. 3). Best back pain (mean (SD)) improvement was observed at 6 weeks (32.1% (32.0)), and then 29.0% (27.8) at 12 months and 25.0% (28.0) at 24 months (decline not significant; Fig. 3 left). Best leg pain improvement was observed at 6 weeks (27.4% (32.0)), and then 23.8% (34.0) at 12 months and 19.1% (32.8) at 24 months (decline not significant; Fig. 3 middle). Best improvement to function was observed at 12 months (17.1% (15.8)), and 15.0% (17.0) at 6 weeks and 15.4% (17.6) at 24 months (decline not significant; Fig. 3 right). The proportion of responders versus non-responders for each subjective outcome was equivalent at yearly time-points (Fig. 3 bottom).

**Table 2:** Mean (standard deviation) values for lumbosacral sagittal alignment, and back pain, leg pain and function

	B	6w	12 m	24 m
LL (°)	53.3 (12.3)	53.3 (10.6)	55.3 (9.2)	
SI (°)	36.4 (7.3)	35.9 (7.0)	36.7 (6.6)	
PDA (°)	9.2 (5.7)	7.0 (4.5)	7.7 (4.8)	
SDA (°)	9.9 (4.9)	9.8 (4.1)	10.5 (4.4)	
RSB (Rmm)	5.6 (38.0)	11.1 (33.7)	9.5 (38.7)	
Back Pain (VAS%)	49.2 (27.4)	17.1 (18.5)	20.3 (21.3)	23.9 (28.0)
Leg Pain (VAS%)	40.1 (33.5)	12.7 (19.4)	16.4 (23.4)	21.0 (30.1)
Function (ODI%)	36.9 (14.7)	21.9 (18.0)	19.8 (16.4)	21.5 (19.6)

*B* preoperative baseline, *6w* 6 weeks, *12 m/24 m* 12/24 months postoperative, *LL* lumbar lordosis, *SI* sacral inclination, *PDA* primary disc angle, *SDA* supradjacent disc angle, *RSB* regional sagittal balance, *VAS* visual analogue scale, *ODI* Oswestry Disability Index

### Outcomes according to responder groups

*Baseline*: Only RSB significantly differed preoperatively between responder groups; subjects with moderate response for function had positive RSB preoperatively (26.7Rmm (42.3Rmm)), while non-responders were negative (−1.0Rmm (32.0Rmm); *p* < 0.05) (Table 3).

**Table 3:** Mean (standard deviation) values at baseline (top) and change at 6 weeks (bottom) for sagittal alignment according to responder group

Case Numbers	Total	Moderate	Minimal	Non
Back Pain	40	17	6	17
Leg Pain	35	13	6	16
Function	40	10	8	22
Baseline				
LL (°)	Back	50.9 (13.6)	54.6 (14.8)	55.2 (10.3)
	Leg	54.2 (14.3)	46.7 (11.5)	54.5 (11.1)
	Function	47.9 (14.6)	56.2 (7.4)	54.8 (11.9)
SI (°)	Back	36.1 (8.4)	36.2 (6.9)	36.7 (6.7)
	Leg	37.1 (8.1)	33.8 (4.4)	36.6 (7.6)
	Function	35.4 (8.5)	33.9 (9.1)	37.3 (6.6)
PDA (°)	Back	8.0 (5.1)	10.8 (4.4)	9.9 (6.6)
	Leg	7.4 (5.8)	9.8 (5.1)	10.2 (5.8)
	Function	7.7 (5.3)	7.0 (5.8)	10.3 (5.8)
SDA (°)	Back	9.3 (5.5)	13.4 (2.4)	9.3 (4.6)
	Leg	10.8 (4.6)	7.0 (6.6)	10.3 (4.5)
	Function	9.6 (4.7)	10.2 (3.5)	10.0 (5.4)
RSB (Rmm)	Back	17.2 (43.3)	2.3 (45.7)	−4.8 (27.1)
	Leg	10.6 (38.3)	17.3 (43.2)	−0.8 (37.0)
	Function	**26.7 (42.3)**	−3.4 (48.1)	**−1.0 (32.3)**
6 weeks – Baseline				
LL (°)	Back	2.6 (11.9)	−1.9 (5.9)	−1.8 (6.5)
	Leg	3.3 (13.2)	−0.7 (3.9)	−1.7 (6.6)
	Function	**5.4 (15.2)**	−0.2 (3.3)	**−2.0 (5.6)**
SI (°)	Back	−0.9 (5.4)	1.6 (3.1)	1.5 (4.2)
	Leg	−1.6 (4.4)	1.1 (3.4)	1.6 (4.9)
	Function	−0.9 (5.1)	−0.8 (4.3)	1.3 (4.5)
PDA (°)	Back	**−0.7 (4.4)**	−1.9 (3.5)	**−3.8 (3.2)**
	Leg	−1.2 (4.4)	−1.7 (2.2)	−3.0 (4.1)
	Function	−0.9 (4.7)	**1.8 (3.8)**	**−3.6 (3.1)**
SDA (°)	Back	0.1 (4.7)	−2.3 (2.2)	0.3 (4.4)
	Leg	1.1 (4.5)	1.0 (4.2)	−0.7 (4.4)
	Function	1.2 (4.7)	−1.3 (4.3)	−0.5 (4.2)
RSB (Rmm)	Back	1.6 (26.2)	−0.3 (13.3)	11.5 (15.2)
	Leg	0.2 (28.7)	**−3.1 (9.1)**	**11.2 (15.5)**
	Function	**−6.2 (31.7)**	3.7 (5.9)	**10.6 (15.0)**

Moderate = responders improving more than 30% in one or more variable; Minimal = responders improving 20–29% for pain and 15–29% for function; *Non* non-responders with less than minimal improvement (or actual deterioration), *LL* lumbar lordosis, *SI* sacral inclination, *PDA* primary disc angle, *SDA* supradjacent disc angle, and *RSB* regional sagittal balance, *Rmm* system relative metric for RSB. Significant differences are indicated in bold (*p* < 0.05)

*Six weeks postoperative change*: PDA flattened by 0.7° (4.4°) in back pain responders while non-responders flattened more (by 3.8° (3.2°); *p* < 0.05). RSB (−3.1Rmm (9.1Rmm)) reduced in leg pain responders, while non-responders increased (11.2Rmm (15.5Rmm); *p* < 0.05). PDA minimally flattened (by 1.8° (3.8°)) in function responders while non-responders flattened more (by 3.6° (3.1°)), while RSB decreased in responders (by 6.2Rmm (31.7Rmm)) and increased in non-responders (by 10.6Rmm (15.0Rmm); *p* < 0.05) (Table 3).

**Fig. 4:**
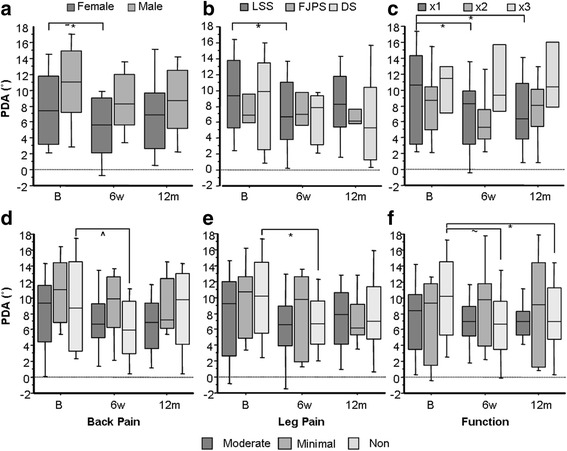
Box-plots revealing serial change in primary disc angle for 40 cases according to sex (**a**), diagnosis (**b**), number of implanted DIAMs (**c**); and responder groups according to back pain (**d**), leg pain (**e**) and function (**f**) (sub-group sample sizes indicated in Table 3). Footnote: *B* preoperative baseline, *6w* 6 week time-point, *12 m* 12 month time-point, *LL* lumbar lordosis, *SI* sacral inclination, *PDA* primary disc angle, *SDA* supradjacent disc angle, and *RSB* regional sagittal balance, *LSS* spinal stenosis, *FJPS* facet joint pain syndrome, *DS* degenerative spondylolisthesis

*Serial change in primary disc angle*: When serial change in PDA was split according to sex, diagnosis, number of implanted DIAMs, and responder groups, differences between sub-groups were noted (Fig. 4). Females (*n* = 20), lumbar spinal stenosis cases (*n* = 27), single DIAM surgeries (*n* = 22), and non-responders in back pain (*n* = 17), leg pain (*n* = 21) and function (*n* = 25) all showed significant early flattening at PDA compared to their sub-group counterparts who were unchanged. Subjects receiving a single DIAM had significantly flatter PDA at 12 months.

### Associations between variables

*Pre*-*operative*: Lumbar lordosis associated with sacral inclination (−0.62, *p* < 0.0001), PDA (−0.48, *p* < 0.01), SDA (−0.54, *p* < 0.001) and RSB (−0.33, *p* < 0.05). PDA was additionally associated with SDA (0.40, *p* < 0.01) and RSB (−0.56, *p* < 0.001). SDA and RSB were weakly associated with function (−0.31, *p* < 0.05) and leg pain (0.42, *p* < 0.01), respectively. Back pain showed a moderate correlation with leg pain (0.54, *p* < 0.001) and function (0.52, *p* < 0.001); leg pain and function associated weakly (0.36, *p* < 0.05).

*Post-operative*: Early changes (6 weeks; Table 4) in RSB and SDA were weakly related to change in symptoms (RSB: back pain (0.33; *p* < 0.05), leg pain (0.40; *p* < 0.05), function (0.43; *p* < 0.01); SDA: leg pain (−0.38; *p* < 0.05), function (−0.35; *p* < 0.05)). Change in lumbar lordosis at 12 months (Table 5) related to change in all other variables, ranging from strong associations with sacral inclination (−0.70; *p* < 0.0001) and SDA (0.64; *p* < 0.0001) to weak associations with PDA (0.40; *p* < 0.05), back pain (−0.40; *p* < 0.05), leg pain (−0.35; *p* < 0.05) and function (−0.32; *p* < 0.05). Relationships between change in back and leg pain and function were equivalent in the early postoperative period (0.67 (back and leg pain), 0.71 (back pain and function), 0.73 (leg pain and function)) than at 1 year (0.49 (back and leg pain); *p* = 0.23, NS); 0.57 (leg pain and function; *p* = 0.47, NS), 0.64 (back pain and function; *p* = 0.30, NS).

**Table 4:** Matrix representing relationships [*r*-values] between sagittal alignment, pain and function according to change at 6 weeks

	LL	SI	PDA	SDA	RSB	BP	LP
SI	**−0.65**						
PDA	**0.49**	**−0.32**					
SDA	**0.57**	−0.15	−0.03				
RSB	**−0.69**	0.20	**−0.52**	**−0.38**			
BP	**−0.37**	0.23	−0.27	−0.24	**0.33**		
LP	−0.26	0.05	−0.19	**−0.38**	**0.40**	**0.67**	
Function	**−0.45**	0.24	−0.24	**−0.35**	**0.43**	**0.71**	**0.73**

*LL* lumbar lordosis, *SI* sacral inclination, *PDA* primary disc angle, *SDA* supradjacent disc angle, and *RSB* regional sagittal balance, *BP* back pain, *LP* leg pain. Statistically significant relationships are indicated in bold (*p* < 0.05)

**Table 5:** Matrix representing relationships [*r*-values] between sagittal alignment, pain and function according to change at 12 months

	LL	SI	PDA	SDA	RSB	BP	LP
SI	**−0.70**						
PDA	**0.40**	−0.20					
SDA	**0.64**	−0.23	0.27				
RSB	**−0.44**	0.06	**−0.52**	**−0.42**			
BP	**−0.40**	0.27	−0.07	−0.08	−0.01		
LP	**−0.35**	0.28	−0.12	−0.14	0.17	**0.49**	
Function	**−0.32**	0.21	−0.22	−0.13	0.14	**0.64**	**0.57**

*LL* lumbar lordosis, *SI* sacral inclination, *PDA* primary disc angle, *SDA* supradjacent disc angle, and *RSB* regional (lumbar) sagittal balance, *BP* back pain, *LP* leg pain. Statistically significant relationships are indicated in bold (*p* < 0.05)

## Discussion

Our study examined sagittal alignment, pain, and function in 40 adults after lumbar surgery augmented with the DIAM, and showed an early postoperative reduction in the index disc angle (by 2.2°) that was not sustained out to 1 year. This subtle and transient change to spinal alignment observed in the postoperative period was apparently unrelated to the improvement in pain and function that was demonstrated at each serial time-point out to 2 years postoperatively. However, preoperative sagittal balance and the early postoperative changes observed for angulation of the index segment were different between patients with and without meaningful improvement. This may point to characteristics of skeletal spinal curvature with prognostic and/or indication-defining potential.

The subtle angulation change to the index segmental angle was expected based on previous biomechanical studies reporting DIAM and other interspinous implants with ex-vivo evidence for an induced posterior element distraction [21, 29] due to reduced posterior disc annular pressure [10], facet joint unloading [30], and limited lumbar extension [20, 21, 29]. In agreement with Sobottke et al. [18], the initial flattening at the index segment (by 3.8° (4.6°) in their DIAM cases) was not sustained and appeared to revert toward preoperative values by 6 to 12 months. This reversion may signify diminished biomechanical effect over time, irrespective of symptoms, which may be secondary to device-settling after resuming habitual upright postures. Our results showing no change to lumbar lordosis and the supradjacent disc angle throughout the postoperative year provide support for a localised mechanical effect at the index segment alone that does not relate to symptoms. Interestingly, and in tacit agreement with our results, Daentzer et al. [13] followed ten patients after implantation with the Wallis interspinous implant, and radiographically showed a subtle reduction to sagittal range of motion of the full lumbar region at their first (up to 10 days; by 3.9°), second (3 months; by 2.8°), and fourth (12 months; by 3.5°) post-operative time-points, yet no statistical difference at 6 or 24 months (approximately 2.5°). It would seem that the skeletal mechanical effects of interspinous implants are short-lived, directing any biomechanical rationale for symptom improvement toward other tissues or physiological processes.

Repeat radiographic evaluation at 24 months was not justified or undertaken in our study for ethical reasons; however, PDA reversion toward baseline at 12 months may indicate a need for longer follow-up wherein non-ionising imaging (like MRI) might be preferable. We showed that non-responders flattened more at the index level than cases who improved, while lumbar lordosis subtly flattened in non-responders yet increased in responders (refer to Table 3 and Fig. 4). This finding suggests that relative kyphosis at either the index segment or lumbar region is not beneficial, which does not support maximising interspinous distraction by inserting the largest device possible as is recommended [9].

As an alternative rationale to altered angulation, the mechanical effect of DIAM in-vivo may instead relate to subtle change to tensile load in local connective and muscle tissues. Further, responders had more positive regional sagittal balance preoperatively that reduced with surgery, while non-responders started negative and became positive. Therefore clinically, preoperative forward trunk inclination might indicate potential for positive response to this surgery, and point to patients that describe relief in flexed postures (a common symptom in lumbar spinal stenosis). The potential influence of a stress-shielding phenomenon wherein soft, or osseous, tissues adapt to tensile loads via Davis’ or Wolff’s Laws, respectively, may provide explanation. Further investigation using appropriate imaging to concurrently examine paravertebral muscles and skeletal recovery under physiological conditions appears warranted to better understand DIAM’s etiology.

Meaningful improvements were observed at 12 and 24 months for back pain and function, while response in terms of leg pain was variable. Therefore, patients describing predominant back pain might be expected to respond better to DIAM-augmented surgery than those with predominant leg pain. However, in assessing absolute change in pain and function we emphasise those with highest preoperative pain scores and limit generalisability to patients with lower levels of pain. Variable pain and function demonstrated by broad standard deviations in our cohort indicate diverse responses that challenge investigators and clinicians to identify patient characteristics leading to best effect. Moreover, it could be argued that lumbar spinal stenosis represents an inclusive diagnosis that requires sub-classification to be meaningful in determining specific response. While our results point to differential diagnosis responses, unfortunately the insufficient power of our sample size did not accommodate multiple sub-group comparisons.

Our results should be interpreted in light of the study limitations. We examined outcomes after DIAM-augmented surgery in a heterogeneous patient group from a single centre. While this may reflect the broad clinical (and global) reality of interspinous implant surgeries, examining discreet patient-groups with fewer covariates would offer improvement. Further, providing suitable distinction between the influences of DIAM in contrast to the surgery it augmented was not addressed and is relevant when equitable outcomes for interspinous implant-augmented decompression versus decompression-alone are shown [3, 16, 18, 31, 32]. As a single-surgeon effectiveness study our sample size was understandably small. However, our findings offer new insights and hint at potential identifiers for patients who may best respond to DIAM (and other) interspinous or lumbar surgeries. Our radiographic imaging was undertaken at several sites for patient convenience. Although routine protocol, it is not ideal in serial analysis of subtle changes to vertebral alignment that is dependent on methodological repeatability and where potential for variable quality exists. Our intra-rater reliability for PDA was low and is indicative of the subtlety of small change to single-segment angulation.

Results of the present study reveal women, cases with lumbar spinal stenosis, or those who receive single DIAM-augmented surgery, show a significant alteration to disc angle at the index segment, albeit brief. Despite change in segmental angulation, its influence on patient’s pain and function appears limited and therefore probably does not explain meaningful subjective improvement. While this study examines objective and subjective measures out to 1 and 2 years, respectively, further investigation of discreet diagnostic categories aimed at isolating the influence of DIAM appear necessary. Sagittal alignment and additional imaging features of functional significance like muscle quality warrant further investigation for interspinous implant or other minimally-invasive surgeries.

## Conclusions

Improvements in back, leg pain and function at 6 weeks and 12 months after DIAM-augmented surgery in patients with varied indications were not reflected in change to skeletal alignment. Subtle radiographic changes at 6 weeks differed according to response, which should be investigated further to identify modifiable risks. Preoperative sagittal balance and posture may have a bearing on outcome.

## Abbreviations

ANOVA: Analysis of variance
DIAM^TM^: Device for intervertebral assisted motion
F: Female
M: Male
NS: Not significant
ODI: Oswestry disability index
PDA: Primary disc angle
Rmm: System-relative measurement for RSB (regional sagittal balance)
SD: Standard deviation
SDA: Supradjacent disc angle

## References

[1] Gazzeri R, Galarza M, Alfieri A (2014). Controversies about interspinous process devices in the treatment of degenerative lumbar spine diseases: past, present, and future.

[2] Wu AM, Zhou Y, Li QL, Wu XL, Jin YL, Luo P, Chi YL, Wang XY (2014). Interspinous spacer versus traditional decompressive surgery for lumbar spinal stenosis: a systematic review and meta-analysis. PLoS One.

[3] van den Akker-van Marle ME, Moojen WA, Arts MP, Vleggeert-Lankamp CL, Peul WC (2014). Interspinous process devices versus standard conventional surgical decompression for lumbar spinal stenosis: cost utility analysis. Spine J.

[4] Lee SH, Seol A, Cho TY, Kim SY, Kim DJ, Lim HM (2015). A systematic review of interspinous dynamic stabilization. Clin Orthop Surg.

[5] Gazzeri R, Galarza M, Neroni M, Fiore C, Faiola A, Puzzilli F, Callovini G, Alfieri A (2015). Failure rates and complications of interspinous process decompression devices: a European multicenter study. Neurosurg Focus.

[6] Siewe J, Selbeck M, Koy T, Rollinghoff M, Eysel P, Zarghooni K, Oppermann J, Herren C, Sobottke R (2015). Indications and contraindications: interspinous process decompression devices in lumbar spine surgery. J Neurol Surg A Cent Eur Neurosurg.

[7] Siddiqui M, Smith FW, Wardlaw D (2007). One-year results of X Stop interspinous implant for the treatment of lumbar spinal stenosis. Spine.

[8] Senegas J (2002). Mechanical supplementation by non-rigid fixation in degenerative intervertebral lumbar segments: the Wallis system. Eur Spine J.

[9] Taylor J, Pupin P, Delajoux S, Palmer S (2007). Device of intervertebral assisted motion: technique and initial results. Neurosurg Focus.

[10] Wilke HJ, Drumm J, Haussler K, Mack C, Steudel WI, Kettler A (2008). Biomechanical effect of different lumbar interspinous implants on flexibility and intradiscal pressure. Eur Spine J.

[11] Crawford RJ, Price RI, Singer KP (2009). Surgical treatment of lumbar segment disease with interspinous implant: review. J Musculoskelet Res.

[12] Roder C, Baumgartner B, Berlemann U, Aghayev E (2015). Superior outcomes of decompression with an interlaminar dynamic device versus decompression alone in patients with lumbar spinal stenosis and back pain: a cross registry study. Eur Spine J.

[13] Daentzer D, Hurschler C, Seehaus F, Noll C, Schwarze M (2016). Posterior dynamic stabilization in the lumbar spine - 24 months results of a prospective clinical and radiological study with an interspinous distraction device. BMC Musculoskelet Disord.

[14] Sur YJ, Kong CG, Park JB (2011). Survivorship analysis of 150 consecutive patients with DIAM implantation for surgery of lumbar spinal stenosis and disc herniation. Eur Spine J.

[15] Mariottini A, Pieri S, Giachi S, Carangelo B, Zalaffi A, Muzii F, Palma L (2005). Preliminary results of a soft novel lumbar intervertebral prosthesis (DIAM) in the degenerative spinal pathology. Acta Neurochirurgica [Suppl].

[16] Kim KA, McDonald M, Pik JH, Khoueir P, Wang MY (2007). Dynamic intraspinous spacer technology for posterior stabilization:case-control study on the safety, sagittal angulation, and pain outcome at 1-year follow-up evaluation. Neurosurg Focus.

[17] Crawford RJ, Malone Q, Price RI (2012). A prospective study for two years after lumbar surgery augmented with DIAM interspinous implant. J Musculoskelet Res.

[18] Sobottke R, Schluter-Brust K, Kaulhausen T, Rollinghoff M, Joswig B, Stutzer H, Eysel P, Simons P, Kuchta J (2009). Interspinous implants (X Stop, Wallis, Diam) for the treatment of LSS: is there a correlation between radiological parameters and clinical outcome?. Eur Spine J.

[19] Crawford RJ, Price RI, Singer KP (2009). The effect of interspinous implant surgery on back surface shape and radiographic lumbar curvature. Clin Biomech.

[20] Phillips F, Voronov L, Gaitanis I, Carandang G, Havey R, Patwardhan A (2006). Biomechanics of posterior dynamic stabilizing device (DIAM) after facetectomy and discectomy. Spine J.

[21] Schilling C, Pfeiffer M, Grupp TM, Blomer W, Rohlmann A (2014). The effect of design parameters of interspinous implants on kinematics and load bearing: an in vitro study. Eur Spine J.

[22] Mannion AF, Balague F, Pellise F, Cedraschi C (2007). Pain measurement in patients with low back pain. Nat Clin Pract Rheumatol.

[23] Fairbank JCT, Pynsent PB (2000). The Oswestry Disability Index. Spine.

[24] Ostelo RWJG, Deyo RA, Stratford P, Waddell G, Croft P, Von Korff M, Bouter LM, de Vet HC (2008). Interpreting change scores for pain and functional status in low back pain: towards international consensus regarding minimal important change. Spine.

[25] Horton WC, Brown CW, Bridwell KH, Glassman SD, Suk S-I, Cha CW (2005). Is there an optimal patient stance for obtaining a lateral 36″ radiograph? A critical comparison of three techniques. Spine.

[26] Spencer P, Steiger S, Cummings H, Genant H (1990). Placement of points for digitising spine films. J Bone Miner Res.

[27] Singer KP, Edmonston SJ, Day RE, Breidahl WH (1994). Computer-assisted curvature assessment and Cobb angle determination of the thoracic kyphosis. Spine.

[28] Kawakami M, Tamaki T, Ando M, Yamada H, Hashizume H, Yoshida M (2002). Lumbar sagittal balance influences the clinical outcome after decompression and posterolateral spinal fusion for degenerative lumbar spondylolisthesis. Spine.

[29] Richards JC, Majumdar S, Lindsey DP, Beaupre GS, Yerby SA (2005). The treatment mechanism of an interspinous process implant for lumbar neurogenic intermittent claudication. Spine.

[30] Wiseman CM, Lindsey DP, Fredrick AD, Yerby SA (2005). The effect of an interspinous process implant on facet loading during extension. Spine.

[31] Marsh GD, Mahir S, Leyte A (2014). A prospective randomised controlled trial to assess the efficacy of dynamic stabilisation of the lumbar spine with the Wallis ligament. Eur Spine J.

[32] Crawford RJ, Lynn JM, Malone Q, Price RI (2012). Pain and function one year after decompressive disc surgery: cases augmented with the DIAM interspinous implant versus those receiving microdiscectomy. J Musculoskelet Res.

